# A rare case of hepatic hilar lymphangioma diagnosed using endoscopic and transabdominal ultrasonography

**DOI:** 10.1055/a-2761-0478

**Published:** 2026-01-13

**Authors:** Koichi Soga, Mayumi Yamaguchi, Masaru Kuwada, Ryosaku Shirahashi, Ikuhiro Kobori, Masaya Tamano

**Affiliations:** 1 26263 Department of Gastroenterology, Dokkyo Medical University Saitama Medical Center, Koshigaya, Japan

Lymphangiomas are rare benign tumors arising from congenital lymphatic system malformations. Intra-abdominal lymphangiomas are uncommon, accounting for less than 5% of lymphangioma cases 1 . Hepatic or hepatoduodenal ligament lymphangiomas are exceedingly rare and of particular clinical interest owing to their anatomical locations.

A 49-year-old woman experienced epigastric pain and nausea early in the morning, followed by pain-induced transient loss of consciousness. She was admitted to the cardiology department with a suspected celiac artery dissection. Subsequent imaging revealed a 4-cm mass in the hepatic hilum; however, a definitive diagnosis could not be established. Abdominal computed tomography (CT) at admission revealed a mildly hyperdense soft tissue area around the celiac artery, without contrast enhancement, suggesting arterial dissection or hematoma. Magnetic resonance imaging (MRI) revealed hematomas of different ages ( Fig. 1 ).

**Fig. 1:**
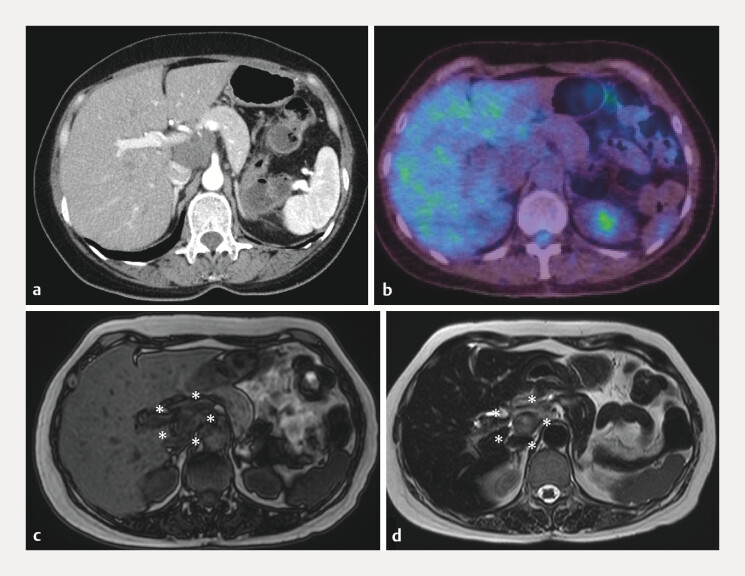
Imaging studies performed for evaluation following emergency admission for epigastric pain. **a** Contrast-enhanced CT (arterial phase) showing a lesion with almost no enhancement, except for a faint linear enhancement within the mass, possibly reflecting septations or hemorrhage. **b** Positron emission tomography-CT showing no abnormal FDG uptake in the lesion with no elevation of the standardized uptake value, suggesting no evidence of malignancy. **c** A MRI T1-weighted image (DIXON opposed-phase) showing the lesion as hypointense, with heterogeneous signal intensity suggestive of an intralesional hemorrhage (asterisk). **d** A MRI T2-weighted image showing a hyperintense lesion with internal septations and fluid components, consistent with hemorrhage within a lymphangioma (asterisk). CT, computed tomography; MRI, magnetic resonance imaging.

No symptom recurrence occurred during the subsequent 3 months. A hepatic hilar mass was suspected as the cause, and the patient was referred to our department for further evaluation. Follow-up CT conducted 3 months later revealed shrinkage of the lesion, excluding the celiac artery dissection ( Fig. 2 ).

**Fig. 2:**
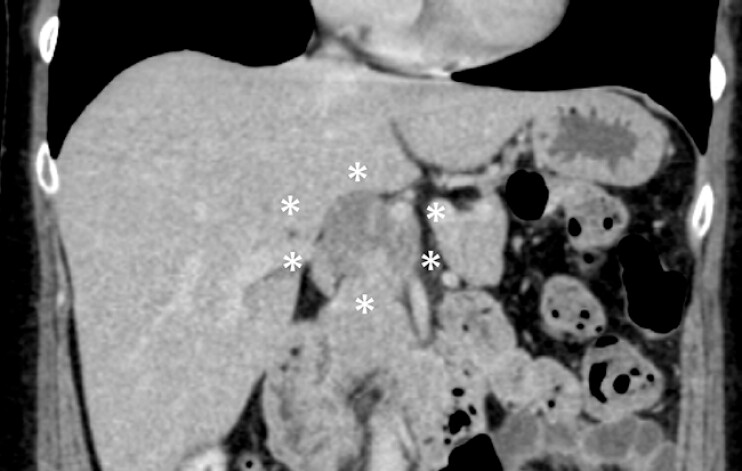
Follow-up contrast-enhanced CT at 3 months following initial presentation. Delayed-phase imaging showing a slight reduction in tumor size, with faint linear enhancement observed along the tumor margin and within the lesion (asterisk). The patient remained asymptomatic without any recurrent abdominal pain during follow-up. CT, computed tomography.

Endoscopic ultrasonography (EUS) revealed a 40-mm mass in the hepatic hilum with internal hyperechoic spots and a marginal solid component believed to be a normal lymph node. Contrast-enhanced EUS using Sonazoid demonstrated minimal contrast within the internal septa, indicating a fibrotic lesion. Based on these findings, a hemorrhagic lymphangioma with secondary degeneration was suspected. Similar findings were obtained on transabdominal ultrasonography ( Fig. 3 , Fig. 4 , Video 1 ).

**Fig. 3:**
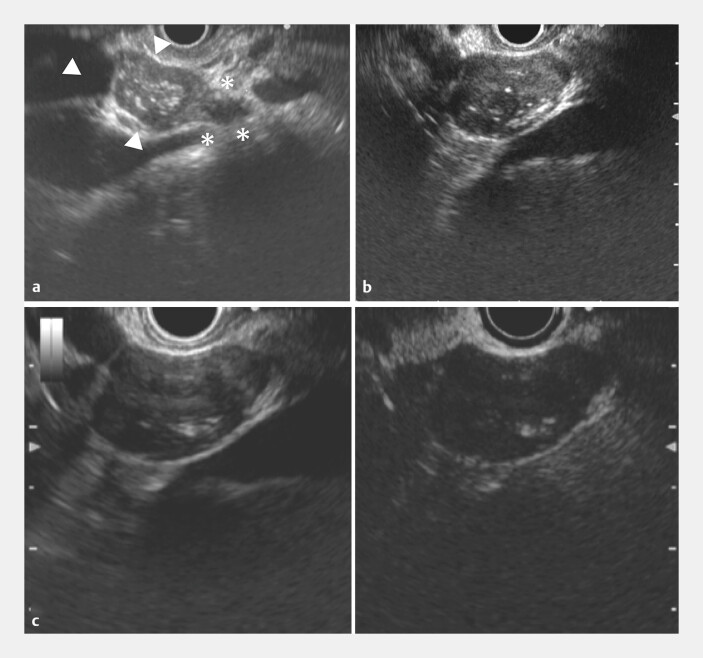
Endoscopic ultrasound (EUS) findings at 6 months after initial presentation. **a** EUS demonstrating a lymphangioma (triangle) arising continuously from a normal lymph node (asterisk). A mosaic pattern of mixed echogenicity, continuous with the lymph node structure, can be observed. **b** A focused EUS view of the lymphangioma showing heterogeneous echotexture with hypoechoic areas and septation-like structures, characteristic of lymphangiomas. **c** left: A conventional EUS image showing internal septation-like structures and cystic changes within the lesion. **c** right: Contrast-enhanced EUS taken using a Sonazoid system showing a linear inflow of contrast medium into the lesion, consistent with the faint linear enhancement pattern seen on delayed-phase CT (following on from Fig. 2 ). CT, computed tomography.

**Fig. 4:**
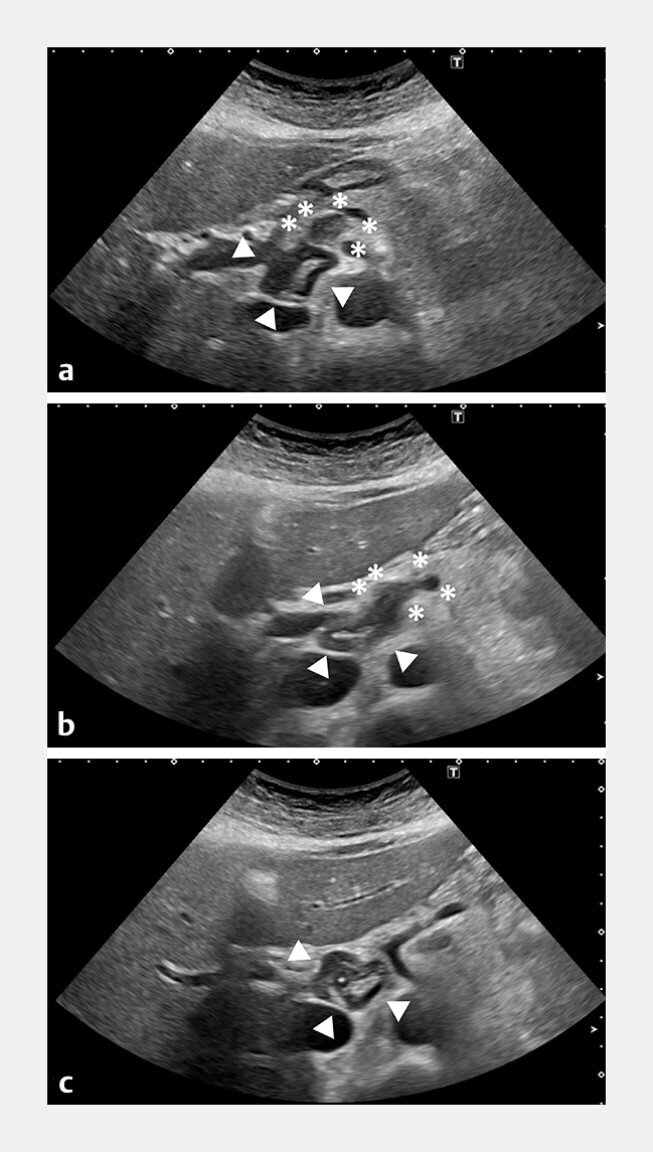
Transabdominal ultrasonography at 6 months after initial presentation. **a–c** Sequential images of the hepatic hilum demonstrating continuity from a lymph node to the lymphangioma. The proper hepatic artery passes through the region surrounded by the lymph node (asterisk) and the lymphangioma (triangle). Panel **a** presents a lymph node and a lymphangioma, panel **b** shows the transition from a lymph node to a mass lesion, and panel **c** depicts the main lymphangioma.

A rare case of hepatic hilar lymphangioma diagnosed using endoscopic and transabdominal ultrasonography.Video 1

The features of lymphangiomas are generally nonspecific, making diagnosis challenging. This case is remarkable as both EUS and transabdominal ultrasonography allowed detailed visualization of a hepatic hilar lymphangioma, which is extremely rare. Contrast-enhanced EUS enabled the noninvasive assessment of the internal tumor architecture, highlighting its potential diagnostic value in characterizing benign cystic or vascular lesions in the hepatic hilum.

Endoscopy_UCTN_Code_CCL_1AF_2AG_3AD

## References

[1] Maghrebi H Yakoubi C Beji H Intra-abdominal cystic lymphangioma in adults: A case series of 32 patients and literature review Ann Med Surg 2022 81 104460 10.1016/j.amsu.2022.104460 PMC9486738 36147158

